# Early onset of breast cancer in British black women

**DOI:** 10.1038/sj.bjc.6604415

**Published:** 2008-06-10

**Authors:** A Cichowska, C M Fischbacher, A Brock, C Griffiths, R Bhopal, S H Wild

**Affiliations:** 1 Public Health Sciences, University of Edinburgh, Teviot Place EH8 9AG, UK; 2 Information Services Division (ISD), NHS National Services, Gyle Square, Edinburgh EH12 9EP, UK; 3 Mortality Statistics, Office for National Statistics, 1 Drummond Gate, London SW1V 2QQ, UK

**Sir**,

We read with interest the article by Bowen *et al* (2008) reporting differences between age of onset, type of breast cancer and survival in British black and white women in East London. They reported that, compared to white women, black women presented on average 21 years younger, that tumours in younger black women were more likely to be aggressive and that survival was poorer among black women despite similar treatment and socioeconomic status.

However, we were surprised by the authors' statement that there have been no previous published data on the patterns of breast cancer in British black women. We recently reported data in this journal on mortality from various cancers including breast cancer by country of birth in the United Kingdom (Wild *et al*, 2006). We used country of birth in our analysis because death certificates in the United Kingdom do not specify ethnicity. Although we recognise that country of birth does not necessarily reflect ethnicity, it is a reasonable proxy. For example 85% of women born in the Caribbean and West Indies identified themselves as ‘Black Caribbean’ in the 2001 Census (Office for National Statistics, 2001).

We reported higher breast cancer mortality, with standardised mortality ratios (SMR) and 95% confidence intervals (CI) among women born in West Africa of 132 (105–163) and in North Africa of 132 (96–176). This was an unexpected finding not reported in previous similar analyses of mortality by country of birth and we were interested to see the potential explanations offered by the findings of Bowen *et al*.

Secondly, we would like to offer an additional explanation for the observed age difference at presentation in the British black women. The national Census indicates that the British black population in the United Kingdom is younger in comparison to the white population (Office for National Statistics, 2001). This may influence the mean age at presentation of disease. The authors have sought to compare the age structure in the two populations; however, because broad age groups (16–59 years and 60 years or more) were used, important variations in age structure may remain unnoticed. It is a common mistake to deduce from the mean age of presentation that the disease occurs earlier. Estimates of age-specific rates are essential to assess whether true differences exist in age at presentation.

Finally, in our study (Wild *et al*, 2006) we did not find a significant excess of breast cancer mortality for women born in the West Indies (SMR 92 [80–106]), the majority of whom are likely to be black. There was a much larger number of breast cancer deaths (208) for women born in the West Indies over the 3-year period of the study than the 85 and 43 breast cancer deaths among women born in West and North Africa respectively. This suggests that there may be heterogeneity of breast cancer risk among British black women. ‘British black’ is likely to be too broad a term for the study of ethnic differences and ethnicity should be further defined where possible (Agyemang *et al*, 2005).
